# Pathology of Cholangiocarcinomas

**DOI:** 10.3390/curroncol30010030

**Published:** 2022-12-26

**Authors:** Nathalie Guedj

**Affiliations:** Department of Pathology, Hôpital Beaujon, 100 Boulevard du Général Leclerc, 92110 Clichy, France; nathalie.guedj@aphp.fr; Tel.: +33-661981787

**Keywords:** cholangiocarcinoma, liver, biliary duct

## Abstract

Cholangiocarcinomas (CCA) are heterogeneous tumors that arise from epithelial cells of the biliary tract. They represent the second primary liver malignancy, after hepatocellular carcinoma. Recent epidemiological data show an increased incidence of intrahepatic CCA without any identified causes. According to their location on the biliary tract, intrahepatic, perihilar (p) and distal (d) CCA can be individualized. Intrahepatic CCA (iCCA) are subdivided into small duct type iCCA and large duct type iCCA, according to the level or size of the biliary duct affected. These two subgroups are characterized by distinct risk factors, gross aspect, histopathological and molecular features, and therapeutic management. The role of biopsy in iCCA is to confirm the diagnosis and to eliminate various differential diagnostics, in particular, metastases. In p/d CCA, biopsy requires more invasive approaches, and tissue samples are difficult to obtain, leading to a high rate of false negatives. In this review, we will discuss the different classifications of CCA (anatomical and macroscopic). We will describe the various microscopic and phenotypic subtypes of CCA. Finally, we will deal with their mode of extension, the role of biopsy and pre-neoplastic lesions.

## 1. Introduction

**Figure 1:**
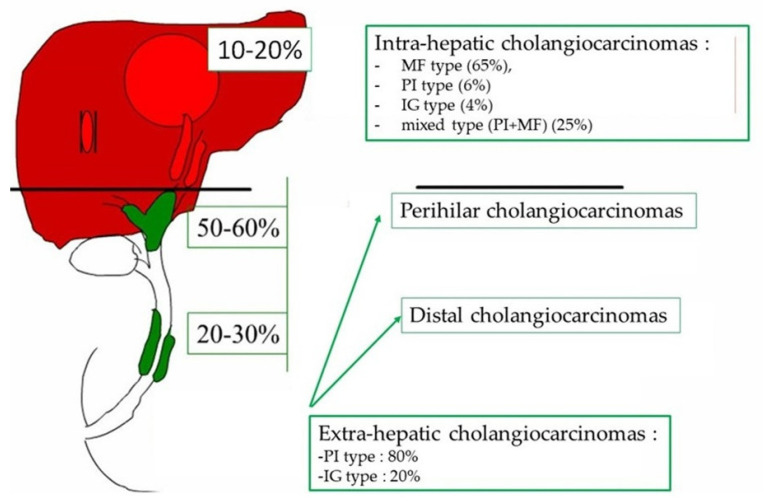
Localization, distribution and frequency of the macroscopic type of cholangiocarcinoma. MF: maa forming, PI: periductal infiltrating, IG: intraductal growth.

Cholangiocarcinomas (CCA) are a heterogeneous group of malignant tumors arising from the biliary epithelium [1]. They represent the second primary hepatic malignancy after hepatocellular carcinoma (HCC). Depending on their anatomical location in the biliary tree, a distinction is made between intrahepatic CCA (iCCA) developed from the intrahepatic bile ducts beyond the second segmentation, perihilar CCA (pCCA) developed at the level of the right and left hepatic ducts, their junction and the hepatic duct and the distal CCA (dCCA) developed at the expense of the common bile duct [2,3]. The last two subtypes fall into the category of extra-hepatic CCA (eCCA) according to the last WHO classification of digestive tumors [3]. Recent epidemiological studies highlight an increase in the incidence of iCCA, of which the causes are not yet identified; meanwhile, the incidence of extra-hepatic CCA has decreased [3]. The frequency of the CCA subtype is unequal and depends on its location. Extra-hepatic forms represent 70 to 90% of CCA while intra-hepatic forms represent 20% (Figure 1). This classification apart from being anatomical is also considered “biological” because each CCA subtype shows a unique biological and pathological profile [1,3,4]. In total, 90% of patients develop sporadic CCA, with no identified risk factor [5]. The known risk factors for developing CCA are chronic inflammation of the bile duct (primary sclerosing cholangitis, hepatolithiasis) and chronic inflammation of the liver (viral hepatitis B or C, steatohepatitis, cirrhosis) [6]. CCA are aggressive tumors, and most patients present at an advanced stage. Diagnosing CCA at an early stage remains a challenge, especially since patients are asymptomatic. Furthermore, the technical difficulty to biopsy the tumor, the abundance of tumor stroma and the paucicellular nature of the tumor limit the sensitivity of cytological and pathological diagnoses. Surgery is the treatment of reference for early-stage tumors, regardless of the anatomical type. However, only 35% of patients are eligible for surgical treatment, and there is a very high rate of post-operative local recurrence. For advanced stages, the treatment of reference is chemotherapy combined with immunotherapy, with a median survival of less than 1 year [2]. New therapeutic modalities that take into account the molecular subtypes of CCA in order to set up individualized targeted therapies are being developed [7]. In this review, we will discuss, from the pathologist’s point of view, the importance of the different macroscopic, microscopic and phenotypic classifications of the different types of CCA. We will also present their mode of extension, their prognostic factors, and the role of biopsy; finally, we will describe preneoplastic lesions.

## 2. Epidemiology and Risk Factors of Cholangiocarcinoma

CCA account for 3% of cancers of the digestive tract worldwide. The peak incidence of CCA is observed during the 6th to 7th decade with a slight male predominance. Due to an appalling prognosis, the mortality rate is roughly equivalent to its incidence rate; the latter is variable and reflects geographical variability mainly linked to the difference in the prevalence of risk factors between populations. The highest incidence rates are observed in the northeast of Thailand (96/100,000 men), China, and South East Asia, while the lowest rate is observed in Australia (0.2/100,000 men) [3,4,6]. In France, the incidence of CCA is 2000 new cases per year, i.e., three times lower than that of hepatocellular carcinoma. iCCA present a different epidemiological profile from p and dCCA with an increase in the incidence and mortality rate of intra-hepatic forms and conversely a decrease in extra-hepatic forms. Over the past 40 years, a steady increase in the age-adjusted incidence rate of iCCA has been observed regardless of the geographical area considered. In the United States, the incidence has increased in 40 years from 0.44 to 1.18 per 100,000 in 2012, i.e., an increase of 2.3% per year and even 4.36% per year over the last ten years. However, these trends need cautious interpretation, given that all versions of the main International Classifications of Diseases (ICD) have failed to include a separate code for the largest group of CCA, and previous versions of ICD have cross-referenced pCCA to iCCA [8]. The decrease in the incidence of eCCA can be explained by the fact that in the databases, cancers of the gallbladder were included in the eCCA groups. However, over the past 30 years, there has been an increase in the rate of cholecystectomy and therefore a decrease in gallbladder cancers [4].

Although a large number of risk factors are clearly associated with the development of CCA, the vast majority (80%) occur sporadically, with no identified etiology [8]. Some of these risk factors are associated with chronic inflammation of the bile ducts, and some are common to iCCA and eCCA (primary sclerosing cholangitis, liver fluke, hepatic lithiasis, congenital biliary diseases), while others are particularly associated with iCCA, in part, those responsible for chronic liver disease (HVB and HVC, alcohol, cirrhosis regardless of the reason, NAFLD, diabetes, NASH).

## 3. Macroscopic Aspects of Cholangiocarcinoma

Macroscopic classification, developed in 1992 by the “Liver Study Group of Japan”, is based on tumor growth criteria and accounts for the biological behavior of the tumor and the prognosis of patients [9]. This classification, allowing for a close correlation with the radiological characteristics, describes three main types of CCA, whether intra- or extra-hepatic: “mass forming”, “periductal infiltratin” and “intraductal growth”. Thus, these different macroscopic types present specific features, in particular microscopic ones, and are differentially observed along the biliary tree.

### 3.1. Mass-Forming Type

**Figure 2:**
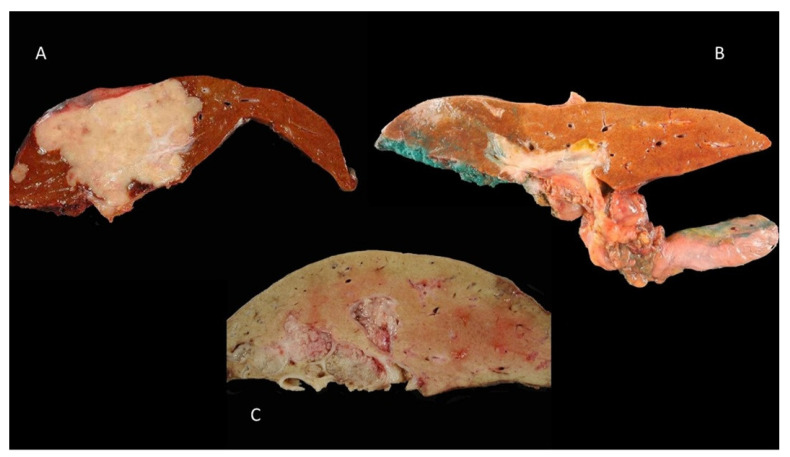
Macroscopic classification of cholangiocarcinoma: (**A**) mass-forming type of an intrahepatic cholangiocarcinoma, (**B**) periductal infiltrating of perihilar cholangiocarcinoma and (**C**) intraductal growth of left biliary duct.

The “mass-forming” (MF) type is defined by a well-limited, rounded intra-parenchymal mass. These tumors are usually firm, whitish, with polylobed contours without a capsule (Figure 2A). This type is mostly seen in small-duct iCCA. The main mass, usually large (mean size 10 cm), is frequently accompanied by centimetric satellite tumor nodules, considered as intrahepatic metastases. These characteristics attest at least in part to the relatively poor prognosis of this macroscopic type. In some cases, there is a retraction of the hepatic capsule next to the tumor.

### 3.2. Periductal-Infiltrating Type

The “periductal-infiltrating” (PI) type is characterized by tumor extension, which infiltrates the biliary wall, producing a diffuse, whitish parietal thickening (Figure 2B). This tumor most often causes a stenosis or an obstruction of the bile duct and is then responsible for a dilation of the bile ducts upstream of the obstacle. pCCA most commonly manifests as indurated bile duct stenosis with infiltration of the periductal and/or adjacent hepatic parenchyma. This type of tumor has a poor prognosis.

### 3.3. Intraductal-Growth Type

The “intraductal-growth” (IG) type concerns a tumoral proliferation developing in the lumen of the bile duct. It is most often revealed at an early stage while the tumor remains localized, polypoid, limited to the mucosa, invading the biliary wall only in the late stages (Figure 2C). In some cases, the tumor is multifocal, involving different segments of the biliary tree and producing a picture of biliary papillomatosis. This tumor type has the best prognosis compared to the other two types, MF and PI.

## 4. Microscopic Aspects of Cholangiocarcinomas

**Figure 3:**
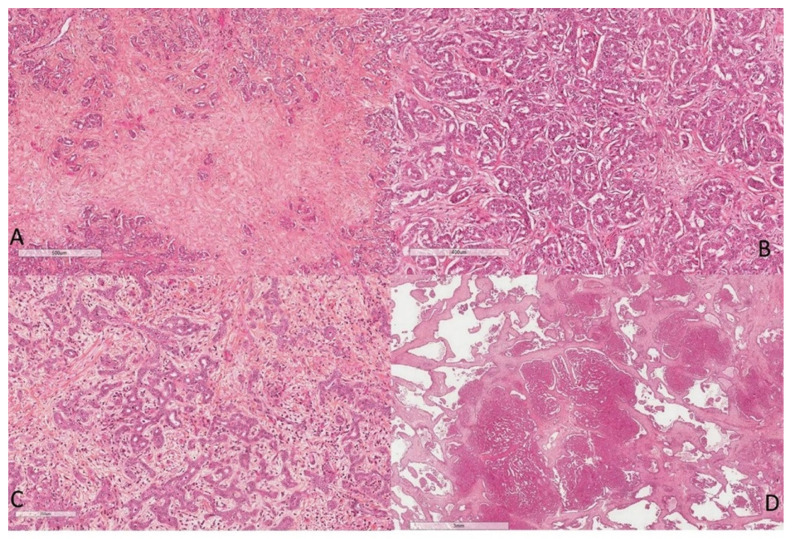
Microscopic aspects of intrahepatic cholangiocarcinoma: (**A**) presence of a desmoplastic stroma, (**B**) small duct type, (**C**) cholangiolocarcinoma, and (**D**) ductal plate cholangiocarcinoma (HES staining ×100).

Most CCA (>90%), whatever their macroscopic type, respond to adenocarcinomas of various differentiation [5]. They present a predominantly glandular pattern in the MF and PI types and a papillary pattern in the IG type. The degree of tumor differentiation is based on the proportion of glandular and/or papillary structures within the tumor proliferation as well as on cytological criteria. CCA are characterized by the presence of an abundant fibrous stroma (Figure 3A), usually with no or little inflammation, harboring numerous blood capillaries [10,11,12]. This fibrous stroma, predominant in the MF type, is one of the main diagnostic criteria of CCA in imaging [13].

### 4.1. Intra-Hepatic Cholangiocarcinoma

iCCA are heterogeneous tumors. Nakanuma et al., have proposed a classification of two main histological subtypes according to the level or size of the bile duct concerned [14]. The small duct type iCCA derives from the intrahepatic small bile ducts (Hering’s duct, bile canaliculus, bile ductules and interlobular bile duct), transformation or transdifferentiation of hepatic progenitor cells or a mature hepatocyte. iCCA are adenocarcinoma with small tubes lined with cubic cells or with acinar architecture, and they infiltrate the hepatic parenchyma (Figure 3B). There is little mucus secretion. Cholangiolocarcinoma, a histological variant of biliary small duct iCCA, is composed of a proliferation of small cells morphologically close to Hering’s duct cells. They are arranged in small regular tubes and are anastomosed, within an abundant fibrous stroma, in continuity at the periphery of the tumor with the non-tumor hepatocyte trabeculae [15,16]. These tumors are very rare (<1% of primary hepatic malignancies) (Figure 3C). Another subtype is biliary small duct iCCA with ductal plate malformation pattern [17]. These are tumors consisting of vague small lobulated nodules with a ductal plane malformation pattern in the center of the tumor and a conventional small duct iCCA at the periphery. The tumor is characterized by large irregular glands, with microcystic dilation, endoluminal fibroepithelial projection and formation of bridges and islets (Figure 3D). The small duct type most often presents as MF.

**Figure 4:**
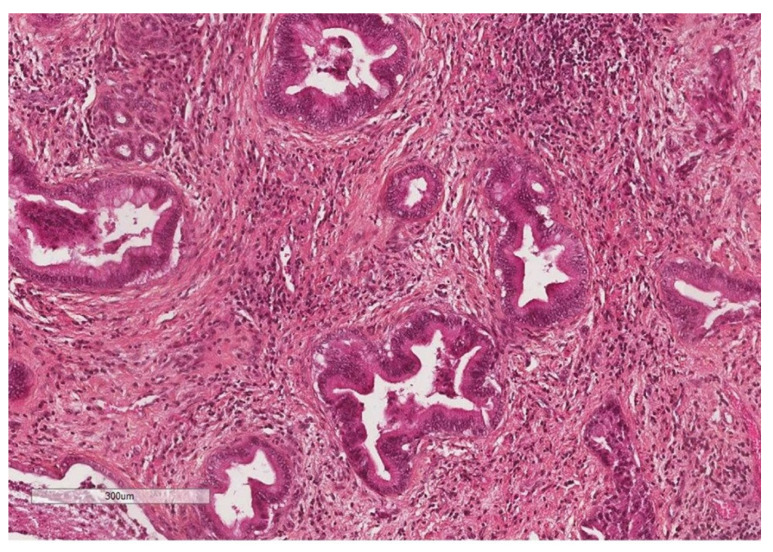
Large duct intrahepatic cholangiocarcinoma (HES staining ×250).

The large duct type iCCA develops at the expense of large intrahepatic ducts (second segmentation) or from the peribiliary glands (Figure 4). These are adenocarcinomas arranged in large ducts or papillae lined by mucus-secreting columnar cells with aspects of invasion of the duct wall and hepatic parenchyma [18]. The large duct type is usually the PI type and less commonly the IG type.

Despite displaying shared mutations in KRAS, SMAD4, ARID1a and GNAS, these two subtypes present distinct clinicopathologic characteristics and molecular profiles. Large duct type iCCA show a high mutation frequency of oncogenes and tumor suppressor genes, such as KRAS (15–30%) and TP53 (10–40%). Apart from these high-frequency mutations, other genes such as BRAF, PI3CA, PTEN, MDM2, EGFR, ERBB2/HER2, PRKACA, PRKACB and many more are mutated. Conversely, small duct type iCCA harbors mutations of IDH1/2 (10–30%) as well as fusions of FGFR2 (10–25%), BRCA ½ (4%) and BAP1 (9–25%) [1,3,18]. In addition, they present different risk factors: cirrhosis, viral hepatitis B and C, steatohepatitis for small duct iCCA and primary sclerosing cholangitis or liver fluke infection for large duct iCCA. Furthermore, large duct iCCA present precursor lesions such as biliary intra-epithelial neoplasia (BilIN) and intraductal papillary neoplasms of the bile duct (IPNB), whereas there is an absence of precursor lesions in small duct iCCA.

### 4.2. Perihilar and Distal Cholangiocarcinomas

**Figure 5:**
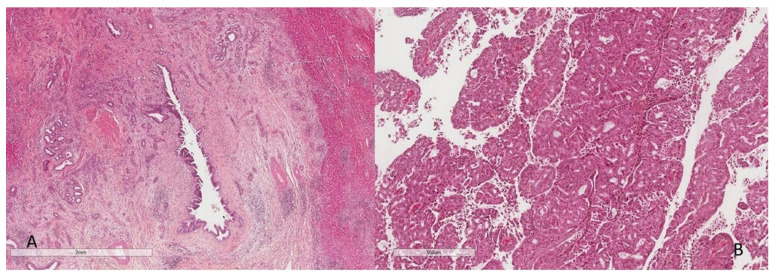
Microscopic aspect of periductal infiltrating (**A**) and intraductal growth (**B**) cholangiocarcinoma (HES staining ×100).

The macroscopic and histological appearance of p/dCCA is similar to large duct type iCCA. They are mainly the PI type and less frequently the IG type. p/dCCA present as a planar or poorly circumscribed sleronodular tumor or less frequently as an intraductal papillary tumor. The vast majority of pCCA and dCCA are conventional mucus-secreting adenocarcinomas. They derive from mucus-secreting columnar cholangiocytes or peri-biliary glands cells, which are also involved at the origin of pre-neoplastic lesions [3,18]. Risk factors and molecular alteration are the same as for large duct type iCCA. However, KRAS mutation frequency seems to be higher in pCCA and dCCA compared to iCCA [18]. In the PI type, carcinomatous proliferation is usually well differentiated, with a tubulo-glandular architecture underpinned by a more or less abundant fibro-hyalin stroma. The carcinomatous structures insinuate themselves between the normal peri-biliary glands along the large-caliber bile ducts (Figure 5A). In the IG type, tumor proliferation is arranged in papillae bordered by more or less differentiated, often large, cylindrical carcinoma cells (Figure 5B). The papillary axes are fibro-congestive and infiltrated with inflammatory cells. These lesions are mainly non-invasive endoluminal proliferations, respecting the basement membrane. When they are invasive, a tubulopapillary architecture can also be observed. In this subtype, extensive sampling of the tumor is essential in order to search for foci of tumor infiltration, which are often microscopic.

### 4.3. Targeted Therapy

The recent discovery of specific molecular alterations of certain CCA subtypes, observed in about 50% of cases, has paved the way for personalized medicine [1,7]. Thus, patients, even in relapse, were able to benefit from a targeted therapy. A large number of genes are currently targetable, such as IDH1/2, FGFR2 and BRCA mutations. IDH1 and FGFR2 mutations are present in 20 and 15% of CCA, respectively [1]. Pemigatinib, a selective FGFR inhibitor, showed a 35% response rate in patients with FGFR2 fusion-positive advanced cholangiocarcinoma in a prospective phase II trial [19,20]. iCCA patients with either MSI-H (frequency 1–2%) or NTRK fusions (1–2%) benefited from treatment with immune checkpoint inhibitors or TRK inhibitors in tumor-agnostic basket trials [21,22]. iCCA seem to benefit more from targeted therapy than eCCA due to a higher prevalence of mutations. The method for detecting an FGFR2 fusion from a FFPE (formalin-fixed paraffin-embedded) tumor sample can be performed either by FISH (fluorescent in situ hybridization) or after extracting DNA by next-generation sequencing (NGS) using multiple gene panels to cover the sequence [23]. IDH and BRCA mutations are equally detected by NGS.

## 5. Immunophenotype of Cholangiocarcinomas

**Figure 6:**
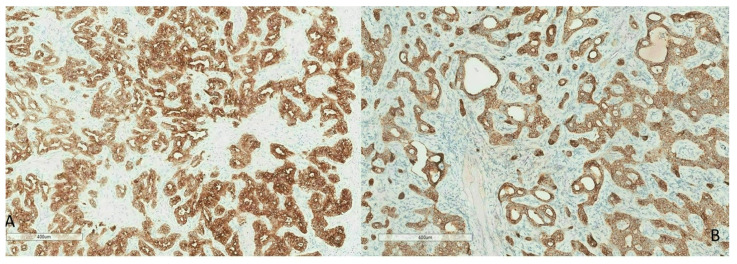
Immunohistochemistry of CK7 (**A**) and CK19 (**B**) showing strong cytoplasmic staining (brown) of an intrahepatic cholangiocarcinoma.

Among epithelial cells of the organism, bile-type epithelial cells exhibit a specific expression profile of cytokeratins (CK). Normal biliary epithelium co-expresses CK7 and 19. During biliary carcinogenesis, malignant epithelial cells continue to express CK7 (Figure 6A) in more than 90% of cases, regardless of their degree of differentiation and CK19 (Figure 6B) in 80 to 90% of cases [24]. However, CK7 and CK19 are not specific markers of CCA since they are expressed by authentic hepatocellular carcinomas [3,18]. According to their immunohistochemical profile, biliary small duct iCCA express CRP, N-cadherin and CD56, while S100P, MUC5AC and MUC 6 are expressed in large duct iCCA [25]. iCCA are of the CK7+/CK20− phenotype while extra-hepatic CCA are often CK7+/CK20+. The more distal the cholangiocarcinoma is in the biliary tree, the more likely it is to be CK20+, with a low to moderate labeling intensity.

## 6. Mode of Tumor Extension and Histoprognostic Factors of Cholangiocarcinomas

Depending on the intra- or extra-hepatic location of the tumor, the mode of tumor extension is different.

### 6.1. Intra-Hepatic Cholangiocarcinoma

iCCA can directly infiltrate the neighboring hepatic parenchyma and reach the portal pedicle and the bile ducts by contiguity. Vascular invasion (branches of the portal vein or hepatic artery) is frequently observed, as well as lymph node extension in the hepatic pedicle. At an advanced stage, intrahepatic metastases are frequent. Various adverse histoprognostic factors are recognized, including the macroscopic type. The 5-year survival of patients with the “intraductal” type is higher compared to that of patients with a “mass-forming” iCCA (69% versus 39%) [4]. Tumor differentiation is a major prognostic factor, with well-differentiated iCCA having a better prognosis than moderately or poorly differentiated iCCA. The presence of a multifocal tumor (main tumor surrounded by satellite nodules), endovascular tumor emboli, lymph node metastases or infiltration of the liver capsule are also poor prognosis criteria. Finally, in the perspective of a surgical treatment, the absence of a resection margin (slice of liver and/or biliary section) is a poor prognostic factor [26].

### 6.2. Extra-Hepatic Cholangiocarcinoma

Extra-hepatic CCA present a mode of extension along the bile ducts, longitudinally and transmurally. Longitudinal extension consists of parietal extension toward the mucosa and submucosa along the biliary tract, with infiltration of the nerve plexuses in more than 80% of cases. This longitudinal extension is difficult to individualize on imaging and requires a frozen section examination of the biliary duct during surgical resections. The mode of transmural extension of the pCCA is responsible for an infiltration of the hepatic parenchyma with direct invasion of the hepatic pedicle and its elements (vein, artery and lymphatics). Extra-hepatic CCA present similar histoprognostic factors to iCCA, including histological type, with a more favorable evolution of the papillary CCA, the grade of histological differentiation, the presence of vascular invasion, perineural invasion, lymph node and portal extension. Similarly, when an extra-hepatic tumor is resected, the incomplete resection (R1) is an important poor prognostic factor [27]. Thus, it has been shown, in a series of pCCA, that the length of biliary resection margin was correlated with the risk of local recurrence. A local recurrence was observed in 18% of patients with a margin of <2.5 mm, 10% with a margin between 2.5 and 5 mm and 0% with a margin of >5 mm [28]. Table 1 shows the main characteristics of CCA according to their location.

## 7. Role of Biopsy in the Diagnosis of Cholangiocarcinoma

### 7.1. Intra-Hepatic Cholangiocarcinoma

The role and the place of the biopsy in the diagnosis of CCA are to be discussed mainly according to the tumor localization. Thus, in case of an intrahepatic mass syndrome, highlighted by imaging, biopsy of the tumor lesion is most often indicated to eliminate differential diagnoses. In practice, the main differential diagnostics of iCCA are hepatocellular carcinoma (HCC) and metastasis of an adenocarcinoma, most often of colorectal origin [29]. Finally, if there are morphological criteria suggestive of CCA in imaging, their formal diagnosis is based on histological examination. The biopsy sample provides tissue material of which morphological and immunohistochemical analyses can most often differentiate iCCA from well-differentiated HCC. HCC has a trabecular architecture and a poor fibrous tumor stroma, which may contain some pseudoacinar structures sometimes centered by bile secretions. The absence of evidence of mucus secretion by Alcian blue staining and the positivity of tumor cells with anti-Hepar or glypican-3 antibodies confirm the diagnosis of HCC. It is more difficult or even impossible to differentiate an iCCA from a metastasis of an adenocarcinoma of the gallbladder, pancreas, or gastric system because the morphological appearance and the tumor immunophenotypic profile (CK7+/CK20−) are very similar. CRP in this context is a diagnostic tool. In iCCA, CRP shows a cytoplasmic staining and has a sensitivity of 93% and a specificity of 88% [30]. In this situation, the clinical data and the imaging assessment in search of a primary tumor are systematically carried out. The most common differential diagnosis is metastasis from colorectal adenocarcinoma. In the case of moderately or poorly differentiated tumors, immunohistochemical study is a diagnostic tool, showing an immunophenotypic profile (CK7−/CK20+) superimposable on normal cells of the colorectal tract.

### 7.2. Extra-Hepatic Cholangiocarcinoma

The role of histology in biliary tract stenosis is initially to distinguish benign from malignant stenosis but also to determine the type of carcinoma. In this context, the pathologist receives two types of samples: a cytological sample and endobiliary biopsies. The problem with these two techniques is the high rate of false negatives. Flow cytometry might increase sensitivity at the expense of specificity. Two more recent techniques are being evaluated: DIA (digital image analysis) and FISH (fluorescent in situ hybridization) [31]. The purpose of these two techniques is to identify aneuploidy indicative of chromosomal instability and therefore of cancer. FISH would be more sensitive than conventional cytology, and the DIA/FISH combination would provide the best values. Finally, the experience of the pathologist in the diagnosis remains the most decisive.

## 8. Preneoplastic Lesions

**Figure 7:**
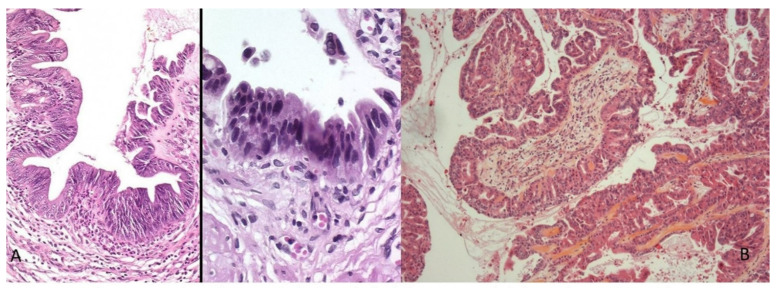
Biliary preneoplastic lesions: (**A**) flat high-grade biliary intraepithelial neoplasia (BilIN); (**B**) low-grade intraductal papillary neoplasia (IPBN) (HES staining ×250).

Similar to other adenocarcinomas, biliary carcinogenesis can follow a sequential process involving preneoplastic or dysplastic lesions. By analogy with pancreatic carcinogenesis, we distinguish, depending on the morphological and functional aspect of the dysplastic cells, two types of neoplastic lesions preceding the invasive CCA [18,20]. Biliary intraepithelial neoplasia (BilIN) is a flat or micropapillary lesion lining the epithelial cells with cytonuclear atypia. It is graded as low or high grade according to the basis of the highest degree of cytoarchitectural atypia (Figure 7A). Various cellular phenotypes are recognized, including biliary, intestinal or gastric. Intraductal papillary neoplasia of the biliary tract (IPN-B) is defined by an atypical biliary epithelium carrying out a papillary growth made up of a conjunctivo-vascular axis and by the production of mucin responsible for dilation of the bile ducts upstream (Figure 7B). Histologically, epithelial proliferation can include four cell types: pancreaticؘ–biliary (MUC1+), intestinal (MUC2+), oncocytic and gastric (MUC5AC+, MUC6+). Morphologically and immunophenotypically, this lesion is similar to intraductal papillary and mucinous tumors of the pancreas [32]. Whatever their type, these preneoplastic lesions occur more frequently in the context of chronic inflammation of the bile ducts, including lithiasis, primary sclerosing cholangitis or parasitic infection.

## 9. Limitations and Future Direction

CCA are highly aggressive biliary tumors with poor prognosis. One of the main limitations in CCA, despite a large therapeutic panel, is that the diagnosis is made at a late stage, which worsens the prognosis of patients. Furthermore, only 20% of patients have a known risk factor. Therefore, it is important to increase awareness of this cancer and promote prevention. Moreover, specific screening policies must be established for the early diagnosis of CCA in high-risk patients [1].

Another limitation is that CCA are clinically, morphologically and molecularly heterogeneous tumors. This heterogeneity represents a limit for the search of a universal treatment but opens up new opportunities for targeted and personalized therapies. Today 50% of patients with CCA have mutations, amplifications and/or fusions that lead to targeted therapy. Scientific research must continue in this direction [8].

One of the characteristics of CCA, whether intra- or extrahepatic, is the presence of a desmoplastic stroma, i.e., rich in collagen fibers. We recently carried out a study on the role of collagen in biliary carcinogenesis, and we were able to show that the cross-linking of collagen fibers is a pejorative prognostic factor in iCCA. LOX A is the enzyme responsible for this cross-linking and could therefore be a new therapeutic target. This example shows that the study of the tumor microenvironment (fibroblasts associated with cancer, immune system, endothelial cells, etc.) represents a major advance in the development of new therapeutic approaches for CCA [10].

## Figures and Tables

**Table 1:** Main charactéristic of cholangiocarcinoma subtype.

Type	iCCA		eCCA
	Small Duct	Large Duct	d + p CCA
Cells of origin	Interlobular bile duct	Peribiliary glands	Cholangiocytes Peribiliary glands
morphology	Small duct with cubic cells and little mucosecretion	Large duct or papillae lined with mucus secreting columnar cells	Same than large duct iCCA
phenotype	Cytoplasmic expression of CK7 and CK19		
	Expression of CRP, N-cadherin and CD56	Expression of S100P, MUC5AC and MUC6	Same than large duct iCCA
extension	High frequency of SN Low frequency of PNI Vascular invasion Metastatic lymph node	Low frequency of SN and high frequency of PNI Hepatic infiltration	Same than large duct iCCA
Molecular alterations	IDH1/2, BRCA1/2 and BAP 1 mutations, FGFR2 fusion	KRAS, TP53 mutations	Same than large duct iCCA with an higher frequency of KRAS mutations

iCCA: intrahepatic CC, eCCA: extrahepatic CC, CK: cytokeratin, CRP: c reactive protein, SN: satellite nodule, PNI: perineural invasion.
